# Development of molecular confirmation tools for swift and easy rabies diagnostics

**DOI:** 10.1186/s12985-017-0853-y

**Published:** 2017-09-22

**Authors:** Kore Schlottau, Conrad M. Freuling, Thomas Müller, Martin Beer, Bernd Hoffmann

**Affiliations:** 1 Institute of Diagnostic Virology, Friedrich-Loeffler-Institut, Südufer 10, D-17493 Greifswald-Insel Riems, Germany; 2 Institute of Molecular Virology and Cell Biology, Friedrich-Loeffler-Institut, Südufer 10, D-17493 Greifswald-Insel Riems, Germany

**Keywords:** Rabies, RT-qPCR, HighSpeed, RT-RPA, Nucleic acid extraction

## Abstract

**Background:**

As rabies still represents a major public threat with tens of thousands of deaths per year, particularly in developing countries, adequate surveillance based on rapid and reliable rabies diagnosis for both humans and animals is essential. Rabies diagnosis relies on highly sensitive and specific laboratory tests for detection of viral antigens. Among those tests, at present the immunofluorescence antibody test is the “gold standard test” for rabies diagnosis, followed by virus isolation in either mice or cell culture. Because of the advantages of molecular assays in terms of sensitivity and applicability their approval as confirmatory diagnostic test by international organizations (OIE, WHO) is envisaged. Therefore, the objective was to develop and validate novel molecular assays and RNA extraction methods for rabies that reduce the turnaround time but remain highly sensitive and specific.

**Methods:**

Here, novel assays, i.e. HighSpeed RT-qPCR and isothermal recombinase polymerase amplification (RPA) were designed and tested. Furthermore, three magnetic bead-based rapid extraction methods for manual or automated extraction were validated and combined with the new downstream assays.

**Results:**

While the conventional column based RNA extraction method showed the highest intra-run variations, all magnetic bead-based rapid extraction methods delivered nearly comparable sensitivity and efficiency of RNA recovery. All newly developed molecular tests were able to detect different rabies virus strains in a markedly reduced timeframe in comparison to the standard diagnostic assays. The observed detection limit for the HighSpeed RT-qPCR was 10 genome copies per reaction, and 1000 genome copies per reaction for the RPA assay.

**Conclusion:**

Magnetic bead-based rapid RNA extraction methods are highly sensitive and show a high level of reproducibility and therefore, are particularly suitable for molecular diagnostic assays including rabies. In addition, with a detection limit of 10 genome copies per reaction, the HighSpeed RT-qPCR is suitable for rapid ante mortem rabies diagnosis in humans as well as confirmatory test in integrated bite management and subsequent post-exposure prophylaxis.

## Background

Rabies is a lethal zoonotic disease caused by a group of 16 negative-strand RNA viruses of the genus *Lyssavirus* in the family *Rhabdoviridae* of the order Mononegavirales [1]. It is a societal tragedy that in the twenty-first century, rabies, a zoonosis that can easily be prevented in humans and controlled in domestic animal species is still neglected and continues to create a significant social and economic burden on a global scale [2–4]. In low-income countries where control efforts are lacking and awareness of the disease and access to appropriate preventive and post–exposure prophylaxis is limited or non–existent, rabies is estimated to still cause around 60,000 human deaths per year [5, 6].

The real impact of this neglected zoonotic disease is likely to be underestimated. One major problem here is surveillance, which unfortunately is inadequate or even non-existing in many Asian and African countries, where the burden of rabies is highest [6–9]. Adequate rabies surveillance in both humans and animals goes hand in hand with rapid and reliable rabies diagnosis. Currently, rabies diagnosis relies on laboratory tests for detection of viral antigens. Among those tests, the fluorescence antibody test (FAT) is the “gold standard test” in post mortem rabies diagnosis [6, 10]. Recently, alternative antigen detection methods such as ELISA, the direct rapid immunohistochemical test (DRIT) or the indirect rapid immunohistochemistry test (IRIT) have been developed which do not require expensive fluorescence microscopic equipment along with the expertise and financial input needed to maintain them [11–15]. The rabies tissue culture infection test (RTCIT) or the mouse inoculation test (MIT) are mainly used as confirmatory tests and based on the complex and time consuming propagation and isolation of the virus [16, 17]. All these test methods, however, are (i) dependent on the quality of the supplied sample, or (ii) require longer turnaround times [6, 18]. Easy to use and swift test systems for rapid, cost-efficient diagnosis, with no loss of sensitivity or specificity would therefore improve the diagnostic situation significantly [12, 18, 19]. In this respect, immunochromatographic strip tests, also called lateral-flow devices (LFDs) or antigen-capture point-of-care tests, have a great potential [20–26], however for rabies there is still a need for standardization and quality check before being an alternative for rapid and simple diagnostics in resource-limited settings [27].

In the course of the past three decades, molecular tools based on the detection of the genetic information of rabies virus have become more widely accepted for the diagnosis of rabies [18]. The development of reverse transcription polymerase chain reaction (RT-PCR) techniques provided an alternative method for post mortem rabies diagnosis [28, 29], and enabled rapid *ante mortem* diagnosis of human rabies [30–32]. Next to conventional or real-time RT-PCRs for rabies [18, 32–37], alternative rapid genome detection tests have been developed, i.e. nucleic acid sequence based amplification and reverse-transcriptase loop-mediated isothermal amplification (RT-LAMP) [34, 38–43]. Other nucleic acid based methods, i.e. HighSpeed RT-qPCR and isothermal recombinase polymerase amplification (RPA), successfully used for detection of other viral pathogens including Schmallenberg virus (SBV), bovine viral diarrhea virus (BVDV), or foot-and-mouth disease virus (FMDV) [44–46] have not been applied for RABV yet. Also, initial efforts to optimize and shorten the process of nucleic acid extraction [47] by remaining highly sensitive and specific have not been further explored.

Therefore, the aim of this study was to (i) design and test both a HighSpeed RT-qPCR and a RT-RPA assay for rapid detection of the RABV, (ii) test the suitability of novel RNA extraction methods that improve reproducibility, analytical sensitivity and operational performance for brain tissue and (iii) combine them with the new downstream assays using different amplification kits. Those tests could then be considered both in advanced as well as in standard laboratories for the detection and confirmation of rabies virus, and thus contribute to the development of better tests for rapid, economical diagnosis for rabies.

## Methods

### Samples

Lyssavirus positive (FAT positive) brain material was provided by the WHO Collaborating Centre for Rabies Surveillance and Research (FLI, Isle of Riems, Germany) comprising field samples and samples from animal experiments (*N* = 12) including the sample subset II (*N* = 43, Table 4) described in a previous study for the evaluation of commercial LFDs for rabies [27]. RABV negative brain tissues (*N* = 8) were obtained from cattle, wolves, sheep and bats.

### Extraction methods

In this study, three magnetic bead-based RNA extraction methods were compared in terms of efficiency to the widely accepted centrifuge-based TRIzol (Life technologies, Darmstadt, Germany) & RNeasy (QIAGEN, Hilden, Germany) method. For each extraction 20 mg of brain tissue was applied.

i) Manual extraction using TRIzol Reagent + RNeasy mini kit

After homogenization of the brain samples with a steel bead in 1 ml TRIzol Reagent (Life technologies, Darmstadt, Germany), chloroform was added and RNA from the aqueous phase was precipitated with 75% ethanol. RNA was further purified with RNeasy Mini Kit (QIAGEN, Hilden, Germany) according to the manufacturer’s instructions and eluted in 50 μl RNase free water.

ii) Manual extraction using the SpeedXtract Virus kit

The brain samples were homogenized with a steel bead in 1 ml PBS and nucleic acids were extracted by the SpeedXtract Virus Kit (QIAGEN, Hilden, Germany) using an adapted protocol with an initial enrichment step. Briefly, 200 μl brain homogenate, 400 μl enrichment Buffer EN (QIAGEN) and 30 μl SpeedXtract Suspension A (magnetic beads) were mixed and incubated. After separation of the magnetic beads in a magnetic stand (Invitrogen), the beads have been washed with 500 μl Buffer EN. Then, the magnetic beads were re-suspended in 200 μl FCPL buffer-master-mixture and lysed by heat (95 °C for 5 min). Finally, 100 μl of the nucleic acid containing supernatant were transferred to a new microtube.

iii) Automated extraction using the EZ1 robotic system

The EZ1 Advanced XL (QIAGEN) is a fully automated nucleic acid extraction. Here, the extraction protocol optimized by Aebischer et al. (2014) [47] was applied. In summary, the DNA Blood Card (QIAGEN) was used, as it represents the shortest extraction protocol (processing time 16 min). For sample lysis, 200 μl homogenized brain sample was mixed 1:1 with buffer VXL (all reagents from QIAGEN). The elution volume was set to 100 μl.

iv) Automated extraction using the KingFisher Duo

Extraction with the KingFisher (KF) Duo platform (Thermo Fisher Scientific, Waltham, MA, USA) was optimized for rapid extraction of blood and serum by Aebischer et al. (2014) [47]. The published protocol has a processing time of only 8 min. The buffers used were identical with the ones of the EZ1 extraction approach but were handled in the 96 deep-well plate format of the KF Duo. The extracted RNA was eluted in 100 μl.

#### Reproducibility of extraction methods

To compare the reproducibility of the standard TRIzol & RNeasy method with the other rapid methods, a single RABV positive brain sample (FLI laboratory ID 21867) was extracted in four biological replicates in three independent runs. All extracted RNAs were analyzed with the standard R14 RT-qPCR [34].

#### Linearity and analytic sensitivity of extraction methods

A ten-fold dilution series of a RABV-positive brain (21867) was prepared. For each extraction method, the samples were tested in duplicates and analyzed with the standard R14 RT-qPCR assay as described below. PCR efficiencies were calculated based on the resulting standard curves.

#### Analytical performance and operational analysis of extraction methods

For analytical performance, eleven RABV-positive archived brain samples from one dog (21,867 (five dilutions)), one horse (1913) and five foxes (1919, 3690, 3695, 14,067, 17,040), as well as one RABV-negative brain were extracted with the four different extraction methods. RABV-specific RNA was then detected with the R14 RT-qPCR assay and the internal control measured in parallel. In order to compare the extraction methods concerning time-to-extract (operational analysis), twelve samples were extracted in parallel and the respective time was measured.

### Amplification and detection methods

For validation of the amplification and detection tests all RNAs were extracted using the manual TRIzol & RNeasy method.

i) Standard RT-qPCR

For amplification and detection of the RABV genome, the standard RT-qPCR assay “R14” was applied [34]. Extraction success was further evaluated using a heterologous HEX-labeled internal control system [48]. The specific RT-qPCR assay was performed using the One-step RT-PCR AgPath Kit (Thermo Fisher Scientific, Waltham, USA). The reaction mix was compromised of 2.25 μl H_2_O, 6.25 μl 2× RT-PCR buffer, 0.5 μl 25× RT-PCR enzyme mix, 0.5 μl primer-probe mix for the internal control and 0.5 μl R14 primer-probe mix. As template 2.5 μl extracted RNA were used. The temperature profile was 10 min reverse transcription at 45 °C, 10 min activation at 95 °C followed by 42 cycles of 15 s 95 °C, 20 s 56 °C and 30 s 72 °C [35]. A BioRad CFX96 Real-time detection system (version 3.1) was used resulting in a run-time of 95 min.

ii) HighSpeed RT-qPCR

The HighSpeed RT-qPCR was developed on the R14-FAM assay, using the same primers and probes [34]. The SensiFAST Probe No-Rox One-step Kit (Bioline, Luckenwalde, Germany) was used with 2.0 μl H_2_O, 5.0 μl 2× SensiFAST Probe Mix, 0.2 μl reverse transcriptase, 0.3 μl RNase inhibitor and 0.5 μl R14-Mix-FAM per reaction, resulting in concentrations of 500 nM RV-N-196-F, 500 nM RV-N-283-R, 125 nM RabGT1-B-FAM in 10 μl of mastermix, which was mixed with 2 μl of extracted RNA. The final temperature profile for the BioRad CFX96 Real-time detection system (version 3.1) was 5 min at 45 °C for reverse transcription followed by 10 s at 95 °C for activation. Then 40 cycles of 1 s at 95 °C for denaturation, and 2 s at 60 °C for annealing, extension and measuring were done. Total run-time was 31 min.

iii) RT-RPA

Based on a RABV MAFFT [49] alignment of all representative full-length sequences of the species rabies lyssavirus available in GenBank, primers and probes suitable for RT-RPA were designed and tested. For amplification the RT-RPA chemistry TwistAmp Exo RT Kit (TwistDx, Cambridge, United Kingdom) was used. The mastermix (22.75 μl) contained 420 nM forward primer RABV-N-71Fv4 (5’ATG GAT GCC GAC AAG ATT GTM TTY AAA GTY AAT AAT CA 3′), 420 nM reverse primer RABV-N-211Rv1 (5′ ATG GAT GCC GAC AAG ATT GTM TTY AAA GTY AAT A 3′) and 120 nM probe RABV-N-196-antisense (5′ TCA AAT CTT TGA TAG CAG GGT ACT TGT ACT CA(FAM-dT) AT(THF) GA(BHQ-dT) CCA CGA TAA TC 3′; FAM-dT = deoxythymidine nucleoside derivated with the fluorophore FAM; THF = tetrahydrofuran; BHQ-dT = deoxythymidine nucleoside carrying a blackhole quencher 1), 14.75 μl rehydration buffer and 5.6 μl RNase free water. The volume for two reactions was applied to rehydrate a single lyophilized reagent pellet, as this contains enzymes and chemicals for a final reaction volume of 50 μl. Afterwards, the rehydrated reaction mix was split into two halves, and the general volume of the reaction was reduced to 25 μl. Finally, 22.75 μl mastermix were mixed with 1 μl of extracted RNA. To start the reaction, 1.25 μl of magnesium acetate (MgOAc) were added. The isothermal reaction was incubated for 15 min at 40 °C in an ESEQuant TS2 instrument (QIAGEN Lake Constance, Stockach, Germany), just interrupted by vortexing the amplification reactions after 5 min. The instrument measured the fluorescence signal in 30 s intervals. Real-time fluorescence data were analyzed using a threshold-based method (ESEQuant TS2 studio software version 1.9.3).

#### Linearity and analytic sensitivity of detection methods

A ten-fold dilution series of extracted and quantified RNA from three different RABV strains (SAD B19, Kelev and Bobcat) was tested with the three different detection methods to determine the analytical sensitivity.

#### Diagnostic sensitivity and specificity of novel molecular detection methods

In order to determine the suitability of the detection methods, a test panel of RNA from field samples (sample subset II [27]) was evaluated in duplicates with the standard method as well as the two rapid detection methods.

### Combination of different extraction methods with novel downstream assays

Eight brain homogenates (one dog (21867), one horse (1913), five foxes (1919, 3690, 3695, 14,067, 17,040) and one negative cow), extracted with four different extraction methods, were tested with three different RABV detection methods to test the suitability of the input material for combined assays for routine diagnostics. Furthermore, the overall analysis time rounded in minutes from sample-to-result was measured.

### Statistics

Regression analysis and the Pearson’s correlation coefficient were calculated to compare the extraction and detection methods. Graphics were made using SigmaPlot v11. PCR efficiencies were calculated using the formula E = 10^(−1/slope)^-1 with the slopes of the standard curves.

## Results

### Rapid RNA extraction methods

Previous unpublished proof of principle tests showed that both published magnetic bead-based automated extraction methods (EZ1 and KF Duo) as well as the new SpeedXtract (SXT) manual extraction kit were all suitable for nucleic acid extraction out of homogenized brain material.

#### Reproducibility of RNA extraction methods

Mean Cq values as well as intra- and inter-run variabilities for the different extraction methods are shown in Table 1. The lowest reproducibility was detected with the standard manual extraction method (TRIzol & RNeasy), whereas the best results were obtained with the manual extraction method SXT. Both extraction instruments delivered comparable results.

**Table 1:** Reproducibility of the standard protocol compared with three rapid extraction protocols. Values were generated out of four extraction replicates in three independent runs. For detection of RABV RNA the R14 RT-qPCR assay was applied

Reproducibility		Intra-run		Inter-run	
Extraction	mean Cq	SD	CV %	SD	CV %
TRIzol & RNeasy	21.23	1.5	6.6%	1.8	8.6%
SpeedXtract	20.82	0.4	2.0%	1.2	5.9%
EZ1	22.87	0.6	2.6%	1.5	6.3%
KF Duo	20.36	0.6	3.0%	1.6	7.9%

SD = standard deviation; CV% = coefficient of variation

#### Linearity and analytical sensitivity of RNA extraction methods

**Fig. 1:**
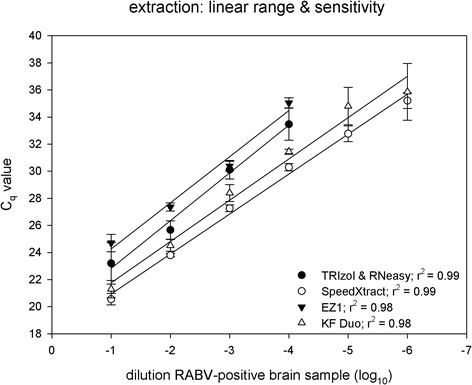
Linearity and analytical sensitivity of the four different extraction methods. A RABV-positive brain homogenate was diluted ten-fold. Numbers indicate mean values for each method. Extracted RNA was quantified with the R14 standard RT-qPCR. R^2^ values are indicated for each method

Analytical sensitivity of the tested extraction methods was determined using a ten-fold dilution series of RABV-positive brain. The manual standard TRIzol & RNeasy extraction method and the EZ1 extraction detected both RABV RNA up to a 10^−4^ dilution, whereas the manual SXT and the KF Duo were more sensitive and detected even the 10^−6^ dilution step. Linearity was good for all four methods indicated by correlation coefficients of 0.98 for the automated extractions and 0.99 for the manual extractions, respectively (Fig. 1). PCR efficiencies were calculated from standard curves of the dilution series. The lowest efficiency (92%) was observed for the standard procedure, followed by the EZ1 method (97%). The KF Duo (113%) and the SXT (118%) yielded comparably higher efficiencies.

#### Analytical performance of RNA extraction methods

**Fig. 2:**
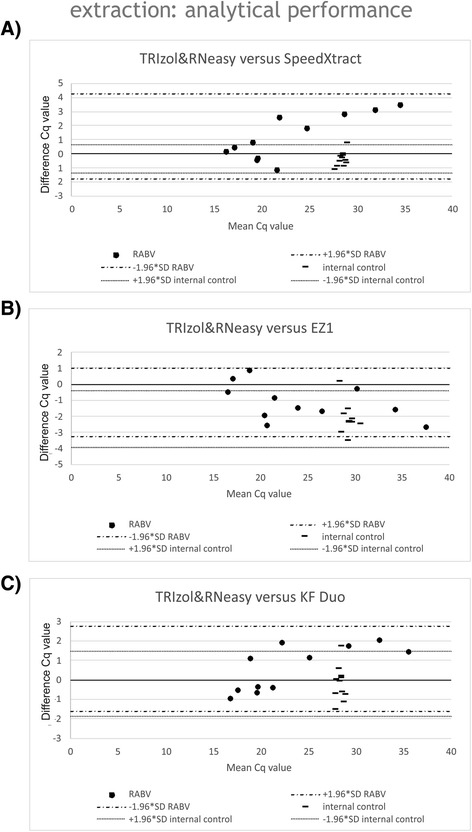
Analytical performance of rapid extraction methods using RABV samples. Ten RABV field samples were extracted using the standard TRIzol & RNeasy method as well as with **a**) SpeedXtract, **b**) EZ1 and **c**) KF Duo. Cq values of the R14 RT-qPCR as well as internal controls were compared

A panel of field samples was extracted to assess the diagnostic sensitivity of the rapid extraction protocols for RABV. The Cq values of the standard TRIzol & RNeasy detection method were compared with the Cq values resulting from the other three methods in a Bland-Altman plot (Fig. 2). The other manual extraction method, SXT, had the lowest correlation to the standard procedure with a coefficient of 0.97. The average Cq value difference was −1.03 (SD = 0.52). Automated EZ1 extraction showed a correlation coefficient of 0.98 and a mean Cq value difference of +0.97 (SD = 0.45), therefore representing a weaker sensitivity. KF Duo also delivered a correlation coefficient for the extractions of 0.98 with a mean Cq value difference of −0.49 (SD = 0.78) (Fig. 2). Furthermore, the heterologous internal control, IC2, was measured in the HEX channel and delivered similar results for three extraction methods, only the EZ1 method showed higher Cq values. The measured mean Cq value with TRIzol & RNeasy was 28.29, for SXT 28.33, for EZ1 30.31 and for KF Duo 28.34.

#### Operational analysis of RNA extraction methods

The time required to extract twelve samples with the four different methods is given in Table 2. The most time-consuming method is the standard manual extraction procedure, which takes about 45 min for the total extraction process including 35 min hands-on time. In contrast, the other three methods require considerably less hands-on time (less than 25 min). The second longest extraction method is the EZ1 procedure taking 41 min. This includes 25 min hands-on time with 15 min thereof dedicated to filling reagent into the cartridge. The KF Duo method takes 24 min in total including 16 min hands-on time. The fastest method was SXT with only 20 min in total and 15 min hands-on time.

**Table 2:** Comparison of hands-on and processing time of four extraction methods evaluated in this study. The time for each operator step corresponds to twelve samples

	Required time (minutes)			
Operator step	TRIzol & RNeasy	SpeedXtract	EZ1	KF Duo
filling of reagents	–	–	15	10
mix sample + lysis buffer	5	5	5	5
incubation	10	5	–	–
load instrument	–	–	5	1
extraction/instrument run	30	10	16	8
total hands-on time	35	15	25	16
total processing time	45	20	41	24

### Rapid amplification and detection using HighSpeed RT-qPCR and RT-RPA assay

Like the standard R14 RT-qPCR assay (amplicon length 87 nt) the two novel rapid detection methods target the RABV N-gene. For the HighSpeed RT-qPCR three different kits (SensiFAST Probe No-Rox One-step Kit (Bioline, Luckenwalde, Germany), Ag-Path ID One-Step RT-PCR kit (Thermo Fisher Scientific, Waltham, Massachusetts, USA), qScript XLT 1-Step RT-qPCR ToughMix (QuantaBio, Beverly, Massachusetts, USA)) were tested. Furthermore, modified temperature profiles were used on three different thermocyclers (BioRad CFX96 Real-time detection system, Illumina Eco Thermal Cycler and Roche LightCycler Nano) to optimize and shorten the existing R14 RT-qPCR. For the RT-RPA the best primer and TwistAmp Exo probe were chosen (amplicon length 140 bp), which led to a reduction of the incubation time. Only the final protocols are given in the material and method section. For better comparability of the results, all Cq values were converted into detection times as RT-RPA is measured in minutes. The total run time of the different amplification methods was 95 min for normal RT-qPCR, 31 min for HighSpeed RT-qPCR, and 15 min for RT-RPA being the fastest method.

#### Linearity and analytical sensitivity of detection methods

The three different amplification and detection methods vary in their sensitivity. The standard R14 RT-qPCR was the most sensitive method. All three RABV strains could be amplified down to one target RNA copy per μl reaction with a good linearity shown by a correlation coefficient of 0.99 for all strains. The HighSpeed RT-qPCR assay was slightly less sensitive with a detection limit of 10 target RNA copies per μl reaction for the strains SAD B19 and Bobcat USA. Only the Kelev strain could be detected with 1 target RNA copy per μl reaction. Also for the HighSpeed RT-qPCR assay an R^2^ value of 0.99 for all strains could be ascertained. RT-RPA amplification yielded the least analytical sensitivity. With this method the determined detection limit was 1000 target RNA copies per μl reaction for all three RABV strains. For the Kelev strain no correlation coefficient could be set, because only two dilution steps were detected. For SAD B19 the R^2^ was 0.94 and for Bobcat USA the R^2^ was 1.0, respectively.

**Fig. 3:**
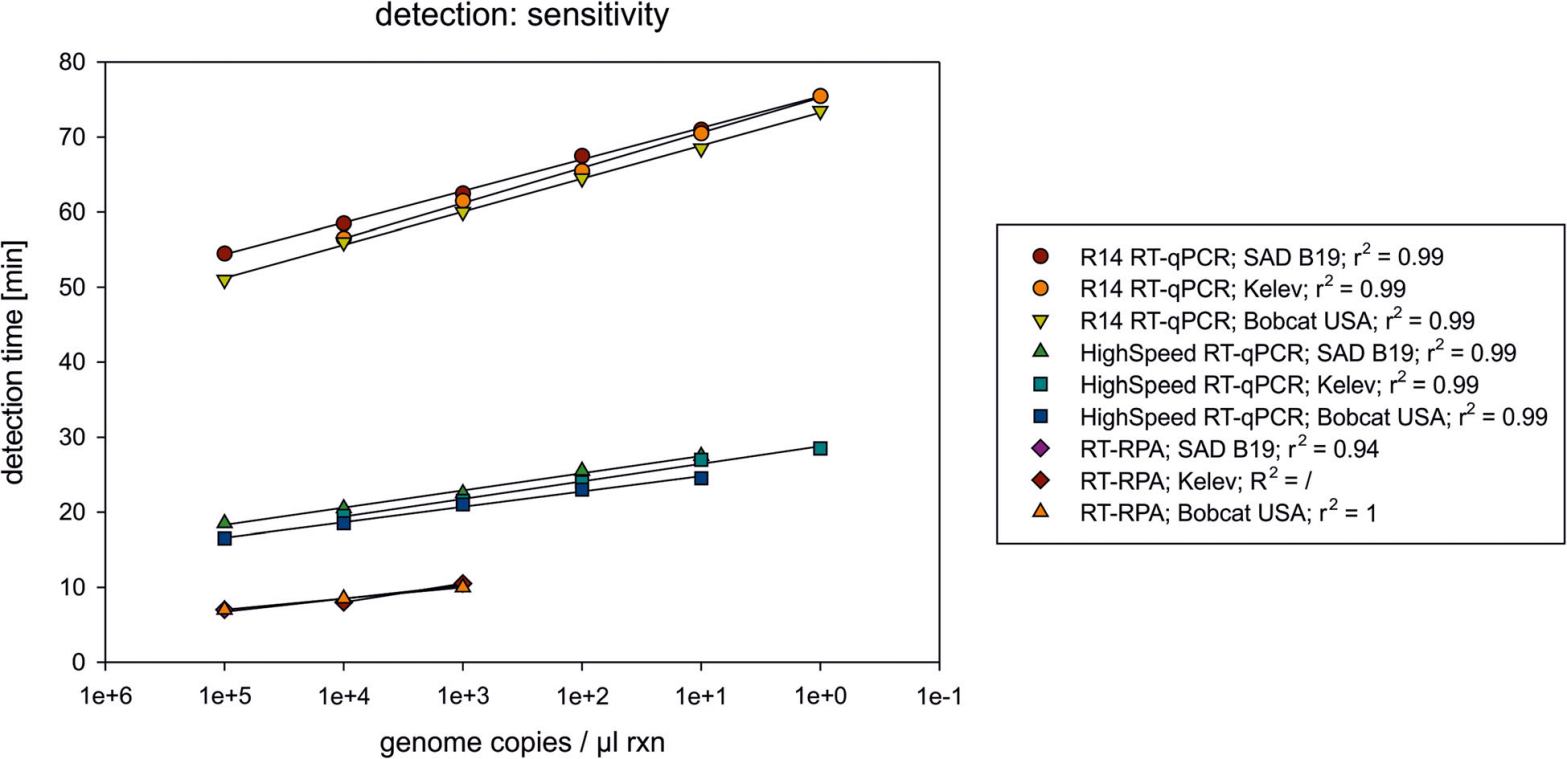
Linearity and analytical sensitivity of the three detection methods. Ten-fold dilution series of three extracted and quantified RABV strains were tested with three different detection methods. Regression lines are illustrated and the correlation coefficient given in the legend

Please note that the starting concentrations of the viral load for SAD B19 and Bobcat USA were 10^5^ copies/μl reaction, whereas Kelev started with 10^4^ copies/μl reaction. A comparison of the detection time of each method reveals a distinct difference between rapid assay (less than 30 min) and standard RT-qPCR (longer than 40 min) (Fig. 3).

#### Diagnostic sensitivity and specificity of detection methods

All three assays detected a broad range of different RABV samples (*N* = 33). The standard RT-qPCR and the HighSpeed amplification method showed a diagnostic sensitivity of 100% (Table 3). The only method showing one false-negative result was the RT-RPA assay, which missed to detect a RABV sample from North America (diagnostic sensitivity 97.0%). The dilution series of RABV sample 20,299 was amplified and detected by all methods. The other tested lyssaviruses showed only one cross detection. DUVV was detected with all three methods with relatively late detection times, whereas EBLV-1, EBLV-2 and BBLV delivered no false-positive results. No amplification of negative samples could be observed.

**Table 3:** Compared detection times of different lyssavirus positive and negative field samples (sample subset II [27]) by measured by R14 RT-qPCR, HighSpeed RT-qPCR and RT-RPA. Cq valued from RT-qPCRs were converted into detection times in minutes (min) to compare them to the detection time of RT-RPA. Numbers are rounded mean values and in brackets given are mean Cq values

Lab ID	Virus species	Species	Origin	Lineage	Detection by Standard R14 RT-qPCR min (Cq)	Detection by HighSpeed RT-qPCR min (Cq)	Detection by RT-RPA min
34,202	RABV	Dog	Yugoslavia	Cosmopolitan	45.0 (15.75)	12.0 (13.80)	5.0
13,491	RABV	Dog	Ethiopia	Cosmopolitan	47.5 (18.03)	13.5 (15.59)	5.5
34,203	RABV	Wolf	Yugoslavia	Cosmopolitan	47.5 (17.90)	14.0 (16.51)	5.0
13,099	RABV	Dog	Taiwan	South-East Asia	48.0 (18.53)	14.0 (16.16)	5.0
13,255	RABV	Human	Chile	Cosmopolitan	40.5 (12.37)	10.0 (11.01)	5.0
8192	RABV	Fox	Bosnia-Herzegovina	Cosmopolitan	51.0 (20.87)	14.5 (17.38)	5.0
3139	RABV	Fox	Germany	Cosmopolitan	48.0 (18.40)	14.0 (16.16)	5.0
13,133	RABV	Cat	Nigeria	Cosmopolitan	50.5 (20.46)	14.5 (17.22)	5.0
13,242	RABV	Bat	South America	American bat variant	52.5 (21.91)	13.0 (15.01)	5.0
13,209	RABV	Mongoose	South America	Cosmopolitan	46.0 (16.63)	12.5 (14.08)	5.0
13,206	RABV	Raccoon	North America	raccoon variant	61.5 (29.16)	24.0 (31.83)	negative
13,200	RABV	Skunk	USA	skunk variant	48.0 (18.38)	14.0 (15.98)	6.0
4131	RABV	Fox	Czech Republic	Cosmopolitan	49.5 (19.48)	15.5 (18.58)	4.5
13,117	RABV	Dog	Algeria	Cosmopolitan	44.0 (15.21)	11.5 (12.59)	5.0
4134	RABV	Fox	Czech Republic	Cosmopolitan	50.0 (19.74)	16.5 (20.29)	4.5
13,056	RABV	Dog	Turkey	Middle East	48.5 (18.68)	13.5 (18.94)	6.0
13,112	RABV	Human	Malaysia	South-East Asia	46.0 (16.85)	14.0 (16.54)	5.0
13,208	RABV	Vampire bat	South America	American bat variant	51.5 (21.31)	18.25 (22.94)	9.0
13,015	RABV	Arctic fox	Norway	Arctic	49.5 (19.73)	13.75 (16.14)	4.5
13,017	RABV	Arctic fox	Norway	Arctic	53.0 (22.50)	15.5 (18.81)	6.0
16,854	RABV	Fox	Kosovo	Cosmopolitan	49.0 (19.01)	13.0 (14.94)	5.5
13,512	RABV	–	South Africa	Cosmopolitan	43.5 (14.89)	10.75 (11.57)	4.5
13,114	RABV	Human	Malaysia	South-East Asia	47.5 (17.90)	12.0 (13.34)	5.0
13,093	RABV	Camel	Emirates	Cosmopolitan	48.5 (18.83)	14.5 (17.45)	8.0
20,299	RABV	Cattle	Iraq	Cosmopolitan	47.0 (17.56)	16.0 (19.52)	5.0
20,299 1:2	RABV	Cattle	Iraq	Cosmopolitan	45.5 (19.20)	12.5 (14.07)	5.0
20,299 1:4	RABV	Cattle	Iraq	Cosmopolitan	50.0 (19.84)	12.0 (13.10)	6.0
20,299 1:8	RABV	Cattle	Iraq	Cosmopolitan	50.0 (20.04)	13.0 (15.14)	5.0
20,299 1:16	RABV	Cattle	Iraq	Cosmopolitan	51.0 (20.74)	13.5 (15.85)	6.0
20,299 1:32	RABV	Cattle	Iraq	Cosmopolitan	52.0 (21.71)	14.0 (16.08)	6.0
20,299 1:64	RABV	Cattle	Iraq	Cosmopolitan	53.0 (22.60)	14.5 (17.15)	6.0
20,299 1:128	RABV	Cattle	Iraq	Cosmopolitan	55.0 (24.22)	15.0 (18.22)	6.0
2498	RABV	Cat	Germany	Cosmopolitan	71.5 (37.19)	23.5 (30.71)	11.0
10,280	EBLV-1	Sheep	experimental	–	negative	negative	negative
10,270	EBLV-2	Sheep	experimental	–	negative	negative	negative
34,494	BBLV	Bat	Germany	–	negative	negative	negative
34,495	BBLV	Bat	Germany	–	negative	negative	negative
12,861	DUVV	Human	South Africa	–	69.0 (35.05)	14.5 (17.40)	5.5
33,341	–	Wolf	Germany	–	negative	negative	negative
33,342	–	Wolf	Germany	–	negative	negative	negative
33,343	–	Wolf	Germany	–	negative	negative	negative
33,344	–	Wolf	Germany	–	negative	negative	negative
33,345	–	Wolf	Germany	–	negative	negative	negative

### Combination of different extraction methods with downstream assays

**Fig. 4:**
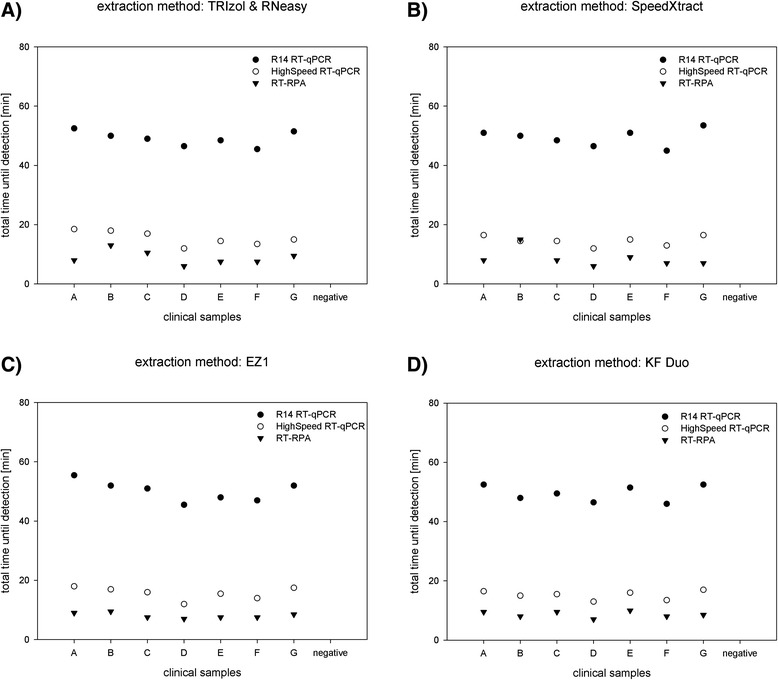
Diagnostic results of eight samples analyzed with R14 RT-qPCR, HighSpeed RT-qPCR and RT-RPA. The previous extraction was made with **a**) manual TRIzol & RNeasy, **b**) manual SpeedXtract, **c**) automated EZ1 and **d**) automated KF Duo. Total time until detection is given in minutes

**Fig. 5:**
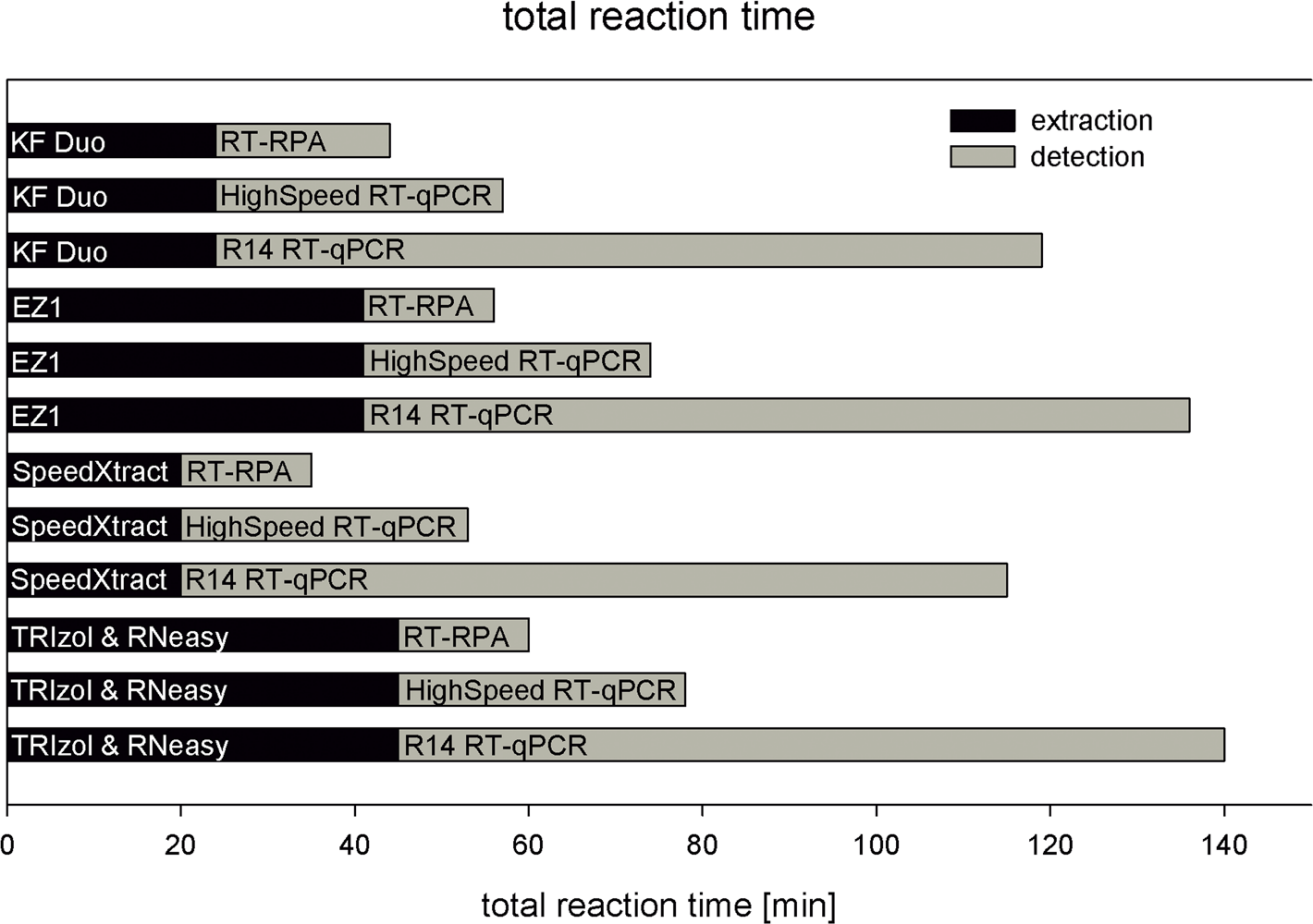
Turnaround time from sample to result for all combinations of nucleic acid extraction methods with amplification and detection methods

Our analysis showed that the final detection results of specific nucleic acid extracts is independent of the application of the downstream amplification assay. The standard R14 assay detected all positive samples between 40 and 60 min. The two rapid assays returned results in less than 20 min (Fig. 4). Consequently, an assessment of the total processing time from sample to result for each combination of methods revealed notably large differences. The routine diagnostic procedure combining TRIzol & RNeasy with R14 RT-qPCR takes more than two hours, whereas SpeedXtract together with RT-RPA takes only about 35 min (Fig. 5).

In addition, the four extraction methods (Table 4) and the three detection methods (Table 5) were compared concerning their suitability as a POCT, for example regarding costs, stability of reagents, robustness, speed and other aspects. Thereby SXT and RT-RPA performed the best.

**Table 4:** Evaluation of different nucleic acid extraction methods in regard to their performance and suitability as POC tests(+++ = good; ++ = medium; + = weak)

	TRIzol & RNeasy	SpeedXtract	EZ1	KF Duo
Equipment costs	++	+++	+	++
Equipment size	++	+++	+	++
Reagent costs	++	+++	++	++
Set up time	+	++	++	++
Total processing time	+	+++	+	+++
Flexibility	++	++	+	+++
Robustness	+	++	++	++
Sensitivity	++	+++	++	+++
Contamination risk	++	++	+++	+++

**Table 5:** Evaluation of different nucleic acid amplification and detection systems in regard to their performance and suitability as POC tests (+++ = good; ++ = medium; + = weak)

	R14 RT-qPCR	HighSpeed RT-qPCR	RT-RPA
Equipment costs	+	+	++
Equipment size	++	++	+++
Reagent costs	++	+++	+
Reagent stability	+	+	+++
Rapid assay design	++	++	+
Reaction speed	+	++	+++
Robustness	+++	+++	++
Sensitivity	+++	++	+
Specificity	++	++	++

## Discussion

Although new techniques have been developed for the diagnosis of rabies in recent years, the number of laboratory confirmed human rabies cases from the most affected countries is limited and represents an underestimate of the real impact of the disease [19, 29]. Therefore, the WHO stresses the need for better tests for rapid and economical diagnosis, with no loss of sensitivity or specificity [6]. The quick and simple to perform diagnostic tests would be ideal for use particularly in countries where laboratory infrastructure is still unfavorable [18, 20]. In the range of diagnostic assays molecular tests are getting more and more attention [18, 29]. Because of the higher sensitivity and specificity over antigen directed assays the molecular nucleic acid amplification tests, i.e. PCRs, are increasingly used as comparative standard detection methods [18, 32–37], although they are not recommended for routine use by international organizations yet [6]. Interestingly, proficiency trials among European laboratories showed that PCRs had less false negative results compared to virus isolation using the standard RTCIT [17].

In this study, we developed and validated specific and sensitive rapid molecular detection methods for the detection of RABV, compared to a published assay and improved the performance of the assays by the combination with novel RNA extraction methods. The results show that simplification of this kind of assays with no relevant loss of sensitivity or specificity can be achieved; thus encouraging both their (i) acceptance as a rapid confirmatory test to first-line assays and (ii) extension of its application in many laboratories, thus improving the overall diagnostic capacities.

However, for detection and amplification of lyssaviral target genomes high quality extraction of RNA is crucial. Manual RNA extractions are prone to errors and relatively time-consuming, but also conventional automated extraction techniques often have lengthy procedures and need trained staff. Recently, automated rapid magnetic bead-based RNA extraction methods have been described but only optimized for blood and serum samples [47]. Therefore, three commercial rapid extraction methods were tested regarding their suitability for brain tissue as the material of choice for *post-mortem* rabies diagnosis and compared to standard extraction method. In terms of reproducibility, the TRIzol & RNeasy method showed the highest intra-run variations (6.6%), and the three rapid extraction methods delivered nearly comparable results. While in the latter case, the EZ1 method as a fully-automated platform was assumed to show lowest coefficients of variation, interestingly best results were obtained with the manual SpeedXtract (SXT) method (2%) (Table 1). Although variations due to variable sample input or samples taken from a different brain region cannot be completely ruled out, sensitivity and efficiency of RNA recovery was acceptable for all rapid RNA extraction methods, with highest rates for SXT as well as KF Duo (Fig. 1). All three tested rapid RNA extraction methods used magnetic particles for nucleic acid extraction but request different instrument equipment. The advantage of the reverse extraction procedure of SXT is that it only requires a heat block and a magnetic stand, whereas the other two rapid methods employ technically sophisticated instrumentation. Both instruments differ enormously in size and weight: the KF Duo is much smaller (40 cm × 46 cm × 34 cm; 17 kg) than the EZ1 (51 cm × 57 cm × 57 cm; 48 kg) making it more attractive for use in smaller laboratories or integration into a mobile laboratory. Regarding the processing time, again SXT and KF Duo performed best (Table 2). The longer total hands-on time for the EZ1 is due to the extensive pre-filling step of cartridges, hence, commercially available cartridges are not suitable for application with shortened extraction protocol. It must be emphasized, however, that the EZ1 method developed by Aebischer et al. includes a self-assembly step, where empty EZ1 cartridges are filled with optimized extraction buffers [47]. Consequently, this EZ1-based method currently is not commercially available and its use depends on the supply of eligible kits (Table 4).

Reduced turnaround times and improved applicability of molecular detection methods can be achieved in different ways. Usually, HighSpeed RT-PCRs take advantage of special but expensive PCR machines with fast heating and cooling ramps, but even with a standard thermocycler (CFX96) an enormous reduction of reaction time for an established detection protocol (R14 RT-qPCR to HighSpeed RT-qPCR) can be achieved.

While the use of thermocyclers depend on a permanent power supply, isothermal amplification methods like RPA can be run, for example, on the ESEQuant TS2; a portable stand-alone, battery powered instrument with multi-channel real-time fluorescence detection capabilities. Highly specific loop-mediated isothermal amplification (LAMP), rolling circle amplification (RCA), strand-displacement amplification (SDA) or helicase dependent amplification (HDA) are either relatively cumbersome in terms of primers design, not rapid enough or costly despite relatively easy primer design and fast amplification [44]. RPA is based on the formation of a recombinase filament, strand displacement and abasic nucleotide analogons and has a broad temperature spectrum [50]. Even successful amplification of target RNA by RPA using body heat has been shown [51]. A further advantage of the RPA method is that in contrast to RT-qPCRs the dried reagents of RT-RPA do not require a cold chain. Unfortunately, the lower analytical sensitivity of RT-RPA (1000 target RNA copies per μl reaction) with a smaller dynamic range seems to run the advantages futile (Fig. 3). This analytic sensitivity seems to be comparable to other described isothermal amplification methods for rabies [39, 42]. However, these methods are proposed as suitable for rabies diagnostics. Although the tested RNAs represent different lyssavirus species and major RABV lineages from different regions of the world, the genetic diversity is still not fully covered and the methods requires further validation. However, at least for the HighSpeed RT-qPCR, which is basically a high speed version of the standard R14 RT-qPCR [34] a combination as multiplex or parallel assays with the R13 RT-qPCR, a slightly modified version of the Wakeley protocol [52], could help to overcome the diversity among RABVs and limit the chance of false negatives tremendously [34].

Despite lower analytical sensitivity, RPA in combination with SpeedXtract would be a promising candidate for simplified molecular diagnosis of rabies. Furthermore, molecular detection methods seem to be more reliable than the diagnostic standard FAT in decomposed animals [53]. As the viral load in brain tissue of rabid animals is relatively high, the methods can aid and enhance the passive laboratory confirmed surveillance under resource-limited settings [27]. Furthermore, the simple test approaches with no requirement for cold chain, could even be integrated into mobile test systems, as shown for food-and-mouth-disease virus or Ebola virus [45, 54] (Fig. 5; Tables 4 and 5).

## Conclusions

Commercial magnetic bead-based rapid RNA extraction methods are suitable for brain tissue and show high sensitivity as well as a high level of reproducibility compared to standard RNA extraction methods and thus, can help standardizing RNA extraction and molecular diagnostics under routine conditions.

Furthermore, the here presented proof-of-principle simplified rapid rabies virus molecular detection methods (HighSpeed RT-qPCR and RPA) showed almost no loss of diagnostic sensitivity or specificity compared to validated standard molecular assays. Thus, the simple, quick and sensitive virus RNA extraction from brain samples combined with fit-for–purpose detection methods may improving the reliability and acceptance for the rapid molecular diagnosis of rabies. Particularly the high analytical sensitivity makes the HighSpeed RT-qPCR a potential candidate as a method of choice as rapid (i) ante mortem diagnostics for human rabies, (ii) differential diagnostics for organ transplantations and (iii) confirmatory diagnostics to first-line assays including integrated bite management and subsequent post-exposure prophylaxis. Especially under resource-limited settings, the SpeedXtract based RNA extraction combined with the RPA detection of the rabies genome could be a useful and robust molecular diagnostic approach.

## Abbreviations

FAT: Fluorescent antibody test
LAMP: Loop-mediated isothermal amplification
LFD: Lateral flow device
PCR: Polymerase chain reaction
PEP: Post-exposure prophylaxis
qPCR: Quantitative/real-time polymerase chain reaction
RABV: Rabies virus
RPA: Recombinase polymerase amplification
RT: Reverse transcription
