# Correction to: Development of molecular confirmation tools for swift and easy rabies diagnostics

**DOI:** 10.1186/s12985-017-0894-2

**Published:** 2017-11-14

**Authors:** Kore Schlottau, Conrad M. Freuling, Thomas Müller, Martin Beer, Bernd Hoffmann

**Affiliations:** 1 Institute of Diagnostic Virology, Friedrich-Loeffler-Institut, Südufer 10, D-17493 Greifswald-Insel Riems, Germany; 2 Institute of Molecular Virology and Cell Biology, Friedrich-Loeffler-Institut, Südufer 10, D-17493 Greifswald-Insel Riems, Germany

## Correction

After publication of the article [1], it has been brought to our attention that an incorrect genetic sequence has been cited. On page 4, paragraph 1 the following sequence is cited “ATG GAT GCC GAC AAG ATT GTM TTY AAA GTY AAT A 3”. This is an error and the correct sequence should be “GGG GGC TTT YCC TAG GGT KAT ACW GGG CTT T 3”.
